# The Antibacterial Protein Lysozyme Identified as the Termite Egg Recognition Pheromone

**DOI:** 10.1371/journal.pone.0000813

**Published:** 2007-08-29

**Authors:** Kenji Matsuura, Takashi Tamura, Norimasa Kobayashi, Toshihisa Yashiro, Shingo Tatsumi

**Affiliations:** 1 Graduate School of Environmental Science, Okayama University, Okayama, Japan; 2 Department of Organismic and Evolutionary Biology, Harvard University, Cambridge, Massachusetts, United States of America; 3 Graduate School of Natural Science and Technology, Okayama University, Okayama, Japan; University of Sussex, United Kingdom

## Abstract

Social insects rely heavily on pheromone communication to maintain their sociality. Egg protection is one of the most fundamental social behaviours in social insects. The recent discovery of the termite-egg mimicking fungus ‘termite-ball’ and subsequent studies on termite egg protection behaviour have shown that termites can be manipulated by using the termite egg recognition pheromone (TERP), which strongly evokes the egg-carrying and -grooming behaviours of workers. Despite the great scientific and economic importance, TERP has not been identified because of practical difficulties. Herein we identified the antibacterial protein lysozyme as the TERP. We isolated the target protein using ion-exchange and hydrophobic interaction chromatography, and the MALDI-TOF MS analysis showed a molecular size of 14.5 kDa. We found that the TERP provided antibacterial activity against a gram-positive bacterium. Among the currently known antimicrobial proteins, the molecular size of 14.5 kDa limits the target to lysozyme. Termite lysozymes obtained from eggs and salivary glands, and even hen egg lysozyme, showed a strong termite egg recognition activity. Besides eggs themselves, workers also supply lysozyme to eggs through frequent egg-grooming, by which egg surfaces are coated with saliva containing lysozyme. Reverse transcript PCR analysis showed that mRNA of termite lysozyme was expressed in both salivary glands and eggs. Western blot analysis confirmed that lysozyme production begins in immature eggs in queen ovaries. This is the first identification of proteinaceous pheromone in social insects. Researchers have focused almost exclusively on hydrocarbons when searching for recognition pheromones in social insects. The present finding of a proteinaceous pheromone represents a major step forward in, and result in the broadening of, the search for recognition pheromones. This novel function of lysozyme as a termite pheromone illuminates the profound influence of pathogenic microbes on the evolution of social behaviour in termites.

## Introduction

Pheromones are involved in probably all social activities including foraging, sexual behavior, defence, nestmate recognition and caste regulation in social insects [1]. Investigating the signalling systems of social insects is essential to understanding their behavioral mechanisms and the evolution of sociality. Therefore, considerable effort has been devoted to identifying their pheromones. The identification of termite trail pheromone provides a well-known example of a great achievement in the history of chemical ecology [2]. However, many important pheromones involved in fundamental social behaviors still remain unidentified. The termite egg recognition pheromone (TERP) has been one of the most important pheromones to be identified [3]–[6]. In addition to the substantial impact in fundamental biology, identification of TERP has considerable economic importance in terms of termite control, because it can lead to an innovative technology [7] for the effective control of the most difficult and economically important pest in the world.

**Figure 1:**
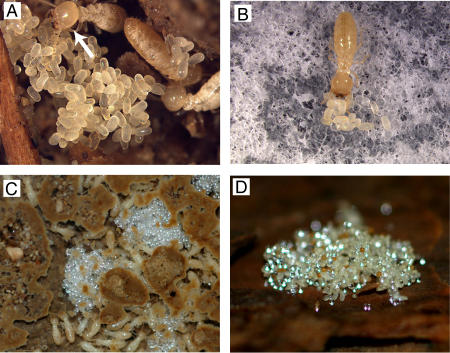
Egg protection behavior in termites. A, Eggs in the nursery cell of *Reticulitermes speratus*. Termite workers always grasp the short side of oval eggs when carrying them. The short diameter provides a critical morphological cue for egg recognition. A worker is shown grooming an egg (arrow). B, Dummy-egg bioassay. Workers bring eggs and egg dummies made of 0.5-mm glass beads coated with the termite egg recognition chemical and form an egg pile in the Petri dish. C, The dummy eggs carried into nursery cells of a semi-natural colony (colony size ca. 500) in the laboratory. Dummy eggs injected in galleries were carried within 48 h. D, Dummy eggs carried into an egg pile in a mature field colony. Dummy eggs were placed in the foraging area of the mature colony. As a result, 6,098 of 31,500 dummy eggs were carried into egg piles in the nursery cells within 48 h.

Egg protection has been known as one of the most fundamental social behaviors in social insects [8]–[10]. In termites, workers recognize the eggs laid by queens and pile these eggs in nursery cells to care for them (Fig. 1A) [3]–[6]. Workers groom eggs frequently and smear their saliva on the egg surfaces to protect them from desiccation and pathogenic infection [3], [6] (see Movie S1). This egg protection behavior is indispensable for egg survivorship [3], [6]. Interestingly, a termite-egg mimicking fungus ‘termite-ball’ was found in *Reticulitermes* termites in Japan and in the US [3]–[6]. Our earlier study demonstrated that termites recognize their eggs based on the TERP and their morphology (size, shape and surface texture) [3], [6]. Egg recognition activity can be tested by a simple bioassay using dummy eggs made of glass beads (Fig. 1B). Glass beads are only carried into the nursery cells and piled with the true eggs when they have been coated with a chemical sample that causes the workers to recognize them as eggs. We confirmed that this bioassay simulated termite behavior in the natural condition, where workers bring the dummy eggs into nursery cells from anywhere in their territory (Fig. 1C, D).

Insect bodies are generally covered with cuticular waxes. The primary function of cuticular lipids is to provide protection from desiccation [11]. In social hymenoptera, it is widely assumed that cuticular hydrocarbons are used in nestmate recognition [12]. In some ants, cuticular hydrocarbons on egg surfaces play an essential role as the egg recognition pheromone, allowing colony members to distinguish between worker- and queen-laid eggs [13] and even between eggs from highly and weakly fertile queens [14]. In termites, however, cuticular waxes extracted from egg surfaces with hexane showed no egg recognition activity [15]. In contrast to ants, termites cannot discriminate eggs from nestmates and non-nestmates [6]. Considering the ecology and evolution of signalling systems, it is reasonable to predict that the egg recognition pheromone may have another practical function, like cuticular waxes which provide egg recognition cues and protect eggs against desiccation in some ants [13], [14]. Eggs of several insect species are known to incorporate noxious components [16] for predator protection or antimicrobial proteins [17] for defence against microbial infection. Our earlier studies suggested that infection of pathogenic microbes is an important lethal factor of termite eggs [3], [4], [6].

## Results

### TERP Is a Proteinaceous Pheromone

**Figure 2:**
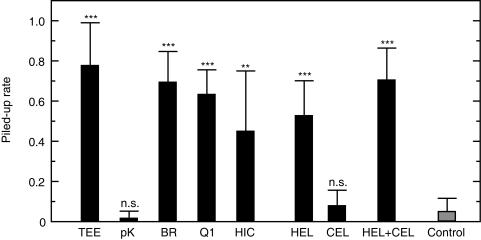
Comparison of the piling rates of dummy eggs made of glass beads coated with test chemicals. TEE: crude termite egg extract; pK: proteinase K digest of termite egg extract; BR: protein samples from the active peaks of BioRex 70 cation-exchange; Q1: UNO Q-1 anion-exchange chromatography; HIC: Methyl Hydrophobic Interaction Chromatography; HEL: hen egg lysozyme; CEL: cellulase. Data are means and standard errors from nine replicates. The chemical concentrations were 1.0 µg/bead for TEE, pK, BR, Q1 and HCI, and 4.0 µg/bead for HEL, CEL and HEL+CEL. Data for each test chemical were compared to the control using two-tailed *t*-tests. **: *P*<0.01; ***: *P*<0.001.

The total lipids extracted from termite eggs by the Bligh-Dyer method [18] had no egg recognition activity (*t* = 0.17, *df* = 16, *P* = 0.86, two-tailed *t*-test), whereas water-soluble extracts showed a strong activity (Fig. 2). Termite workers perform egg protection behaviors even toward the eggs of different species indiscriminately, at least in the genus *Reticulitermes*, suggesting a broad cross-species activity of TERP (Table S1). To determine whether TERP is a protein or other water-soluble substance, we digested termite egg extracts with Proteinase K (Nacalai Tesque, Inc., Kyoto). After proteinase digestion, the egg extracts completely lost egg recognition activity (Fig. 2). The cationic proteins separated by cation-exchange with BioRex 70 resin (BioRad Laboratories, CA) showed significant activity (Fig. 2). These results clearly indicate that TERP is a protein, not a lipid, in contrast to social hymenoptera.

### Isolation and Molecular Size Determination of the TERP

**Figure 3:**
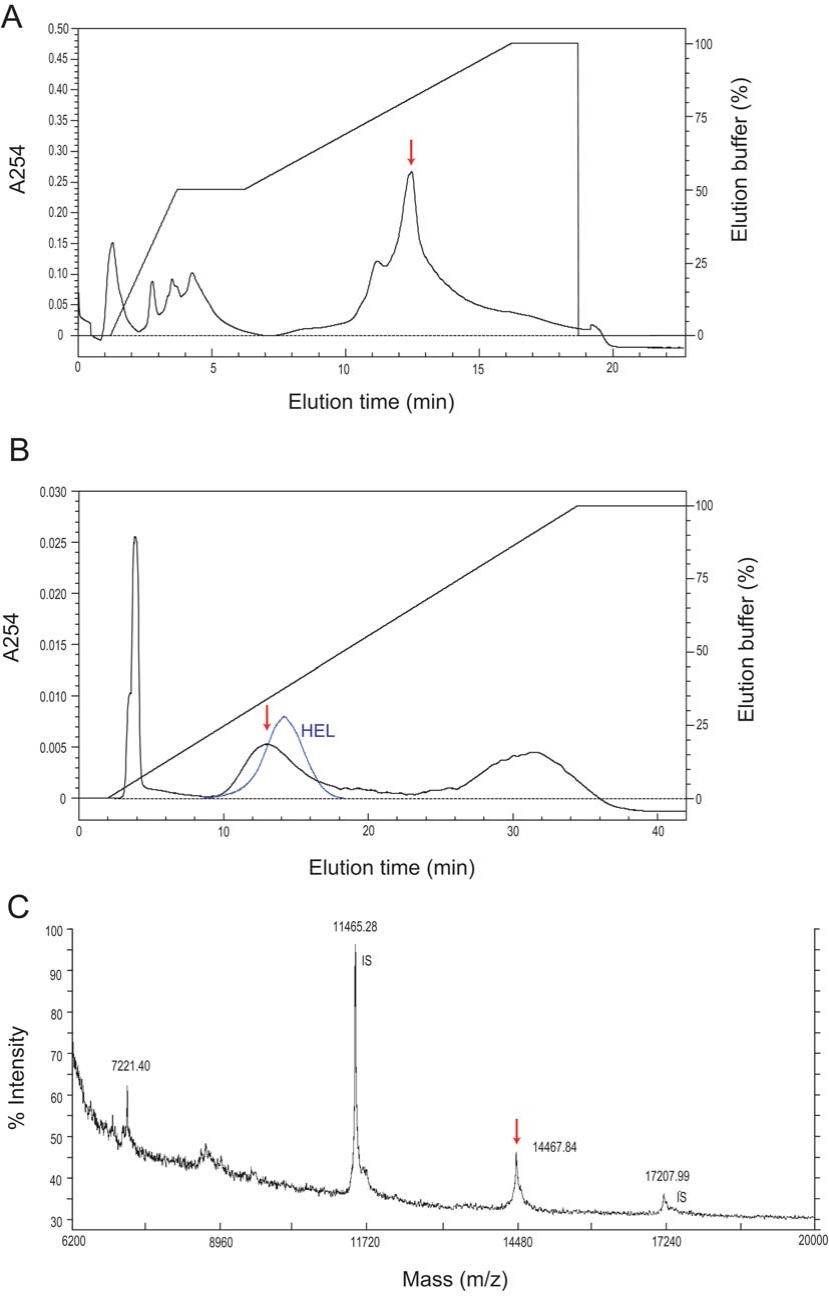
Chromatography and MALDI-TOF MS analyses. A, UNO Q-1 anion-exchange chromatography elution patterns of the TERP. The active peak is indicated by an arrow. Lysozymes bind with coexisting anionic proteins in the Tris-HCl buffer (pH 8.2) and thus are eluted as the absorbed fraction, although lysozyme itself is a cationic protein. B, Methyl HIC column chromatography. The active peak is indicated by a red arrow. The elution pattern of hen egg lysozyme (HEL) is indicated by the blue line. C, MALDI-TOF MS spectrum of the purified TERP. Ions at m/z 14467 and 7221 (divalent ion) were identified as the target protein. Ions at m/z 11465 and 17207 were the dimer and trimer of insulin used as the internal standards (IS), respectively.

For a rough estimate of the molecular size of the TERP, the proteins were separated by ultra-filtration with NMWL 50,000, 20,000, 5,000 and 3,000 filter membranes. The TERP passed through the NMWL 20,000 filter membrane, but did not pass through the NMWL 5,000 membrane, suggesting that the TERP is a protein with a molecular size between 5 and 20 kDa. Gel filtration chromatography showed an active peak around the 17 kDa peak of the molecular standard (Fig. S1). The TERP was purified to a single active protein through steps involving BioRex 70 cation-exchange and chromatography using UNO Q-1 and Methyl HIC columns (Fig. 3A, B). The MALDI-TOF MS analysis confirmed the purity of the target protein and showed that the molecular mass of the TERP was 14.5 kDa (Fig. 3C). Importantly, we found that the termite egg extract provided antibacterial activity against a gram-positive bacterium *Bacillus subtilis* (NBRC 3009; Fig. S2). Among the currently known antimicrobial proteins, the molecular size of 14.5 kDa limits the target to lysozyme.

### Antibacterial Protein Lysozyme Is the TERP

**Figure 4:**
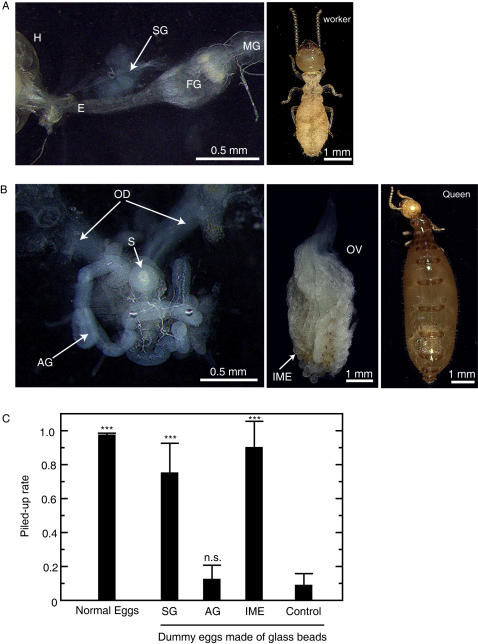
Worker and queen organs producing the egg recognition pheromone. A, Salivary gland (SG) of a *Reticulitermes speratus* worker. H: head, E: oesophagus, FG: foregut, MG: midgut. B, Accessory gland (AG) and ovary of a queen. OD: oviduct, S: spermatheca. C, Egg recognition activity of the extracts from worker salivary glands (SG), queen accessory glands (AG) and immature eggs collected from a queen ovary (IME). We compared the piling rates of dummy eggs made of glass beads coated with these extracts. The chemical concentrations were 0.025 worker equivalent extracts (eq.) per bead, 0.03 queen eq. and 0.02 queen eq., respectively. Data are means and standard errors from fifteen replicates (5 replicates × 3 colonies). Data for the three colonies were pooled because there was no significant difference among the colonies. Data for each treatment were compared to the control using two-tailed *t*-tests. ***: *P*<0.001.

Lysozyme (EC 3.2.1.17), an antibacterial enzyme, is found in many cells, tissues and secretions from a large range of organisms including mammals, birds, reptiles, fishes and insects [19]. Lysozyme hydrolyses the β1,4-glycosidic linkage between N-acetylmuramic acid and N-acetylglucosamine of bacterial peptideglycan. Lysozymes have been characterised in several insects. Insect lysozymes have been reported to defend against bacterial infection or aid in digestion [20]. In termites, lysozymes have been identified from worker salivary glands and the digestive tract, and have been regarded as digestive enzymes [21]. The antimicrobial compounds in saliva play a major role in pathogen defence in termite colonies [22]. It seems reasonable that the chemical compounds of saliva also function as the TERP, since termite eggs are frequently groomed by workers and are thereby coated with saliva (see Movie S1 and Fig. S3). To examine the egg recognition activity of termite saliva, we extracted salivary glands from workers and conducted egg recognition bioassays (Fig. 4A). Glass beads (0.5 mm) coated with the salivary gland extract showed highly significant egg recognition activity (Fig. 4C). We purified termite lysozymes from egg extracts and salivary gland extracts by cation-exchange column chromatography following the insect lysozyme purification protocol [23]. Both the termite egg lysozyme and the salivary lysozyme showed significant lytic (Fig. S2) and egg recognition activities (termite egg lysozyme: *t* = 5.532, *df* = 22, *P*<0.0001; salivary lysozyme: *t* = 4.12, *df* = 22, *P*<0.001).

Lysozymes have been classified into several distinct types based on amino acid sequences and tertiary structures [19]. Hen egg lysozyme (14.3 kDa) is the most frequently studied protein. Termite lysozyme (NCBI Accession No.: BAC54260) shows approximately 40% amino acid homology with hen egg lysozyme (1AZF) and both are classified as c-type lysozymes. The phylogenetic tree of amino acid sequences for c-type lysozymes is shown in Fig. S4. The commercially available hen egg lysozyme (SIGMA, St. Louis) showed a strong termite egg recognition activity (*t* = 7.69, *df* = 16, *P*<0.0001; Fig. 2). The proteins and peptides used as negative controls (bovine serum albumin, myoglobin, insulin and cellulase) showed no activities by themselves (*df* = 16, *P*>0.05, two-tailed *t*-test). These results clearly showed that the TERP was the antibacterial protein lysozyme.

Lysozyme is characterised by a very high isoelectric point, about 11.0, enabling it to bind readily to other coexisting proteins and cause precipitations. After aggregation, lysozyme loses both enzyme and pheromone activity. In our research, the termite egg extracts became easier to precipitate and lost their activity during storage as purification proceeded (Fig. S1), while crude egg extracts maintained their activity for a long time. Various chemicals besides lysozyme are present on egg surfaces and in termite saliva. Some of these coexisting chemicals may act as supporting chemicals, stabilizing the activity of lysozyme in natural conditions. Cellulase (EC 3.2.1.4), a well-known component of termite saliva [24], showed no egg recognition activity by itself (*t* = 1.24, *df* = 16, *P* = 0.23). However, the addition of cellulase (Sigma, St. Louis) to lysozyme significantly promoted egg recognition activity compared to lysozyme alone (*t* = 9.25, *df* = 16, *P*<0.0001; Fig. 2).

### Lysozyme Gene Expression in Termite Eggs

**Figure 5:**
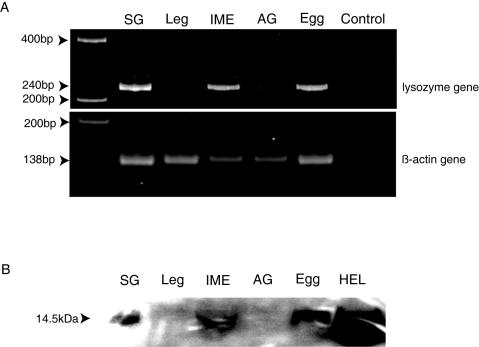
RT-PCR and western blotting for lysozyme gene expression analysis. A, RT-PCR of mRNAs extracted from worker salivary glands (SG), worker legs, immature eggs dissected from queen ovaries (IME), queen accessory glands (AG) and eggs collected from termite nests. B, Western blot analysis with the anti-lysozyme polyclonal antibody performed on the protein extracts from each tissue and hen egg lysozyme (HEL) as a positive control.

To be recognized as eggs by workers after being laid by queens, eggs should already be covered in TERP at oviposition. In other words, eggs must have lysozyme on their surface before they are first groomed by workers. This suggests two possibilities: (I) lysozyme is produced and secreted by eggs themselves or (II) lysozyme is produced by accessory glands of queens and eggs are coated with the accessory gland secretion, or both. We found a strong egg recognition activity in the extract of immature eggs collected from queen ovaries, but no significant activity in the extract of queen accessory glands (Fig. 4B, C). To investigate the transcription of lysozyme genes in eggs and accessory glands, we performed reverse transcript (RT)-PCR analysis using mRNA isolated from the salivary glands and legs of workers, the accessory glands of queens, immature eggs collected from queen ovaries and oviposited eggs collected from a natural colony. The integrity of the mRNAs was verified by RT-PCR using primers for the universal housekeeping gene ß-actin [25]. This amplification yielded bands of the expected size (138 bp) from mRNA extracted from each tissue. The termite lysozyme specific primers yielded the expected 240 bp bands from mRNAs extracted from salivary glands, immature eggs and oviposited eggs (Fig. 5A). This result shows that lysozyme genes are expressed in eggs and worker salivary glands. Two allozymatic lysozymes (Lys1 and Lys2) have been reported in termite salivary glands [21]. We amplified and sequenced the cDNAs coding each allozymatic lysozyme expressed in eggs and salivary glands. We found expression of both types of lysozyme in eggs and salivary glands. The cDNA sequences of Lys1 (GenBank Accession No.: EF198324) and Lys2 (EF198326) expressed in eggs were identical to those of Lys1 (EF198325) and Lys2 (EF198327) expressed in salivary glands, indicating that termite egg lysozymes and termite salivary lysozymes are identical. Furthermore, we performed western blot analysis using a polyclonal rabbit anti-lysozyme antibody to confirm the production of lysozyme in immature eggs and oviposited eggs. The 14.5 kDa bands were detected in immature eggs and oviposited eggs as well as salivary glands, whereas no lysozyme band was detected in legs and accessory glands (Fig 5B).

## Discussion

Since Fleming discovered lysozyme in 1922, it has been subject to extensive investigation as a model protein [19]. Hen egg lysozyme was the first protein sequenced and subjected to X-ray crystallographic analysis. Despite a huge body of information, the biological function of lysozyme is still not completely understood. All of the known digestive and defence functions are attributed to its lytic activity. However, in this study, we found a novel function of lysozyme as an insect pheromone, where the signalling function seems independent of the enzymatic activity. Our findings have implications for the evolution of chemical signalling systems. It is relatively common for pheromone substances to have additional practical functions such as chemical defense [26]. Termites use their saliva not only in food digestion but also in some other important behaviors including grooming, burying corpses and nest construction. Therefore, lysozyme exists everywhere in termite nests. This may explain why termites do not rely purely on chemical cues but also use morphological cues for egg recognition [3], [6].

The nesting and feeding ecology of termites exposes colony members to a great variety of microbes including bacteria, fungi, protozoa, viruses, spirochetes and nematodes [27]. In this sense, we can say that termites live in the world of microorganisms [4]. Therefore, one of the most important selection pressures on termites is how they cope with various microorganisms, resulting in the evolution of behavioral and physiological adaptations [4]. Researchers have focused on the ecological and evolutionary significance of diseases and disease resistance mechanisms of social insects to better understand the selective pressures that have influenced their social organization and the evolution and maintenance of insect sociality [27], [28]. Our finding that termites use their antibacterial chemicals as releaser pheromones for social behaviors indicates the evolutionary linkage between anti-pathogenic adaptations and social behaviors. Over the last two decades, researchers have focused almost exclusively on hydrocarbons when searching for recognition pheromones in social insects. Thus, the present finding of a proteinaceous pheromone may represent a major step forward in, and result in the broadening of, the search for recognition pheromones in social insects.

In addition, considering applied biology, termites are one of the most economically important pests in the world and one of the most difficult to control. The annual economic cost of termites may be as high as $11 billion in the United States alone [29]. *Reticulitermes* termites are classified as multiple-site nesters based on their nesting and feeding habits [30]. Multiple-site nesting makes finding the reproductive cells and controlling these insects very difficult [31]. Based on the present findings, artificial objects could be readily introduced into the royal central cells using egg-carrying behaviors. Dummy eggs filled with pesticides could be introduced into the royal centre of a colony, destroying the entire colony with only a small amount of pesticide [7]. Our identification of the TERP may lead to such technical innovation in termite control in the near future.

## Materials and Methods

### Preparation of Termite Eggs

The workers, eggs and queens used in this experiment were collected from *Reticulitermes speratus* colonies in Okayama and Kyoto, Japan. In total, we used 1,708,400 eggs (71.75 g) in the chemical analyses and bioassays. To examine cross-species egg recognition activity, we used another 12 species collected in Japan and the United States (Table S1). Soil and wood dust were removed from eggs using termite egg carrying behaviors. The eggs collected from the wild nests of *R. speratus* were placed on a moist unwoven cloth on a 90-mm Petri dish together with 100 workers and kept at 25°C for 24 h. Workers carried eggs and formed egg piles in the Petri dish. Termite balls were carefully removed from the egg piles using forceps under a stereoscope. In this way pure termite eggs were obtained for chemical analyses and bioassays.

### Egg Recognition Bioassay

Dummy-egg bioassays were performed in 35-mm Petri dishes (Corning, NY) using workers from three *R. speratus* colonies collected in Okayama, Japan. Dummy eggs were made of 0.5-mm diameter glass beads and were coated with each test chemical. The diameter of the glass beads matched the short diameter of termite eggs. The test chemicals were dialysed with BioDesign Dialysis Tubing (MWCO 3500, BioDesign Inc. NY) against distilled water and lyophilised to remove buffer salts before the bioassay because buffer salts interfere with egg recognition. Each test chemical was re-suspended in 30% glycerol ddH_2_0 to a final concentration of 0.05 mg/µl, and then 2 µl of the solution was added to 100 glass beads. Glycerol was used to keep the test chemicals on the surface of the glass beads. Glycerol itself had no egg recognition activity. Ten eggs and 20 dummy eggs were randomly arranged on moist unwoven cloth in a Petri dish, and 10 workers were released in the dish. Dishes were maintained at 25°C in the dark. The acceptance rates were determined by counting the number of dummy eggs carried onto egg piles after 24 and 48 h. This bioassay was replicated three times for each of the three colonies unless otherwise stated; data for the three colonies were pooled because there was no significant difference in egg carrying activity among the colonies (Two-way ANOVA, colony: *F*_(2,12)_ = 0.50, *P* = 0.62, treatment: *F*_(2,12)_ = 51.77, *P*<0.00001). Data were arcsine-root-transformed before statistical analysis. The mean piled-up ratio of each treatment was compared with the control (only 30% glycerol) using two-tailed *t*-tests.

### Extraction and Purification of the TERP

Termite eggs were homogenised by grinding with a mortar and pestle, and diluted 1∶10 (w/v) in ddH_2_O. The egg solution was centrifuged for 30 min at 15,000 rpm at 4°C. The supernatant was collected and lyophilised, and then used as a crude extract. Lipids were removed from the crude egg extract using the modified Bligh and Dyer method [18], and then 30 mg of crude egg extract were dissolved in 3 ml of 1∶2 chloroform/methanol and vortexed, and 1 ml of chloroform was added and vortexed. Finally, 5 ml of ddH_2_O were added and vortexed. The mixture was centrifuged for 30 min at 4000 rpm at 4°C. The chloroform layer containing lipids was evaporated on a rotary evaporator until all of the solvents were removed. The lipids were re-suspended in chloroform to a final concentration of 0.05 mg/µl. A 2-µl sample was then added to 100 glass beads and the solvent was evaporated. The upper phase (methanol-water layer) containing non-lipids was collected and used in the next purification procedure.

To examine whether the TERP was a protein or other water-soluble substance, we digested termite egg extracts with Proteinase K (Nacalai Tesque, Inc., Kyoto). The crude extract was diluted 1∶30 (w/v) in ddH_2_O and digested with 2 mg/ml proteinase K at 37°C for 24 h. For a rough estimate of the molecular size of the TERP, the termite egg extracts were separated by ultra-filtration with Amicon Ultrafree-MC (NMWL 50,000, Millipore, Bedford, MA), Centricut mini V-20 (NMWL 20,000, Krabou, Osaka), Amicon Ultrafree-MC (NMWL 5,000, Millipore, Bedford, MA) and Microcon YM-3 (NMWL 3,000, Millipore, Bedford, MA).

#### Bio-Rex 70 cation exchange

The egg extract solution was applied to Bio-Rex 70 cation exchange resin (BioRad Laboratories, CA) equilibrated with 50 mM phosphate buffer, pH 6.8. The column was washed with the same buffer and the adsorbed protein was eluted with 0.5 M NaCl in the same buffer. The active fraction was pooled, dialysed (MWCO 3500) against distilled water and lyophilised.

#### Gel filtration column chromatography

The lyophilised protein obtained from the active fraction of the Bio-Rex 70 cation exchange was applied to the sequence of gel filtration chromatography, anion exchange chromatography and hydrophobic interaction chromatography (HIC) using the BioLogic DuoFlow Chromatography System with the BioFrac fraction collector (BioRad Laboratories, CA).

To roughly estimate the molecular weight of the target protein, gel filtration chromatography was performed on the active fraction obtained by cation exchange. The lyophilised sample was redissolved in 50 µl of 50 mM phosphate buffer (pH 6.8) with 0.15 M NaCl and applied to a Bio-Silect SEC 125 Gel Filtration Column (7.8 mm×300 mm; BioRad Laboratories, CA) equilibrated with the same buffer. Fractions of 0.5 ml were collected at a flow rate of 1.0 ml/min. The active fractions were pooled, dialysed (MWCO 3500) against distilled water and lyophilised.

#### UNO Q-1 anion exchange chromatography

The lyophilised protein obtained from the active fraction of the Bio-Rex 70 cation exchange was redissolved in 1.0 ml of 20 mM Tris buffer, pH 8.2, and centrifuged for 5 min at 15000 rpm. The supernatant was applied to a UNO Q-1 anion exchange column (7 mm×35 mm; BioRad Laboratories, CA) equilibrated with 20 mM Tris buffer, pH 8.2. The absorbed protein was eluted with a linear gradient of NaCl concentration from 0 to 0.5 M in the same buffer, at a flow rate of 2 ml/min. The active fractions were pooled and then dialysed (MWCO 3500) against distilled water, because buffer salts interfere with egg recognition. The sample was then lyophilised for use in the egg carrying bioassay.

#### Hydrophobic Interaction Chromatography

The lyophilised protein from the previous step was redissolved in 1.0 ml of 100 mM phosphate buffer, pH 6.8, with 2.4 M ammonium sulfate. The sample was applied to a 5-ml Econo-Pac Methyl HIC column (BioRad Laboratories, CA) equilibrated with the same buffer. The protein was eluted with a linear gradient of ammonium sulfate concentration from 2.4 to 0 M in the 100 mM phosphate buffer, pH 6.8, at a flow rate of 2 ml/min. The active fraction was then desalted using the Bio-Gel P-6 Desalting Gel (BioRad Laboratories, CA), following the manufacturer's instructions, and then lyophilised. The egg recognition bioassay for HIC samples was replicated three times using a single colony due to the limited amount of the sample.

### Matrix-assisted Laser Desorption Ionization-time of Flight Mass Spectrometry (MALDI-TOF MS) Analyses

To examine the purity of the target protein and to obtain a molecular mass, MALDI-TOF MS was performed. The purified target protein from the previous step was dissolved in 50% acetonitrile. Insulin was added to the sample for an internal standard. The sample (1.5 µl) was applied to a stainless steel MALDI target plate (Applied Biosystems, USA), overlaid with 0.5 µl of a saturated matrix solution of α-cyano-4-hydroxy cinnamic acid (αCHCA), and dried at room temperature. The average molecular mass of a single-charged ion of the target protein was determined using the Voyager-DE STR MALDI mass spectrometer (Applied Biosystems, USA) equipped with a nitrogen laser (337 nm). Mass spectra were acquired in the linear positive-ion mode by using an accelerating voltage of 25,000 V, a grid voltage of 90%, and an extraction delay time of 300 ns. Calibration mixture (ACTH [clip 18–39], insulin, and apomyoglobin) was used as external standards. Spectra were obtained by accumulating 500 consecutive laser shots. Data were analysed using Data Explorer software version 4.0 (Applied Biosystems).

### Lytic Activity of Termite Lysozyme

The termite egg lysozyme (TEL) and termite salivary gland lysozyme (TSGL) were purified from termite egg extract and salivary gland extract according to the insect lysozyme purification method [23]. The lytic activity of lysozyme was measured against 0.2 mg/ml *Micrococcus lysodeikticus* (Sigma, St. Louis) suspended in 0.1 M phosphate buffer (pH 6.8). Two micrograms of lysozyme extract were added to 3 ml of the reaction buffer. Two replicates were made of each treatment. The decrease in absorbance at 450 nm was measured with a SmartSpec Plus Spectrophotometer (Bio-Rad Laboratories, Hercules, CA).

### Extraction of mRNA and Reverse Transcript (RT)-PCR

To determine the biochemical origin of lysozyme, we investigated the transcription of termite lysozyme genes. We extracted mRNA from the salivary glands (SG) of 40 workers, the legs of 20 workers, the immature eggs (IME) from one queen's ovaries, the accessory glands (AG) of eight queens and 15 mg of eggs collected from the termite nests using the Nucleospin RNA II total RNA isolation kit (Macherey-Nagel Inc., PA), following the manufacturer's instructions. Pure RNA was finally eluted in 60 µl RNase-free water. RT-PCR was performed using the QIAGEN OneStep RT-PCR Kit (QIAGEN Inc., CA) following the manufacturer's protocol. The reverse transcription reaction was performed at 50°C for 30 min, and the mixture was incubated at 95°C for 15 min to activate *Taq* DNA polymerase.

It has been reported that two types of allozymatic lysozyme (Lys1 and Lys2) are expressed in termite salivary glands [21]. To investigate the expression of lysozyme genes regardless of allozymatic variation, we designed the primers TL-F (5′-ATGGACGTGAGAAATTCTCCG-3′) and TL-R (5′-KATCTGGAACAGGCCATAATC-3′) from a highly conserved nucleotide sequence found in cDNA of Lys1 and Lys2, which amplifies a 240-bp partial termite lysozyme gene. PCR was conducted using 35 cycles of 30 sec at 94°C, 10 sec at 52°C, and 15 sec at 72°C. The cycles were followed by incubation for 10 min at 72°C. To check the contamination of genomic DNA, reverse transcription was omitted in the negative control.

To verify the integrity of the mRNA used in this experiment, samples were amplified by PCR using the housekeeping gene ß-actin primers [25] (forward, 5′-AGAGGGAAATCGTGCGTGAC-3′; reverse, 5′-CAATAGTGATGACCTGGCCGT-3′; resulting fragment length, 138 bp; conditions: initial denaturation at 95°C for 15 min, followed by 35 cycles; denaturation at 94°C for 30 s, annealing at 60°C for 5 s, and extension at 72°C for 10 s). Amplified products were electrophoresised on a 12% polyacrylamide gel at 100 V for 90 min, then stained with ethidium bromide and visualised by UV illumination. A low DNA mass ladder (Invitrogen, CA) was included in each run. The molecular size of each band was analysed using the Lane and Spot Analyzer version 6.0 software (ATTO, Tokyo, Japan).

### Sequencing the Termite-egg Lysozyme cDNA

To compare the sequences of lysozyme coding cDNAs expressed in eggs and those expressed in salivary glands, we amplified and sequenced the cDNAs coding each allozymatic lysozyme. We amplified a 360-bp partial sequence of Lys1 coding cDNA (Rs-Lys1) and a 559-bp partial sequence of Lys2 coding cDNA (Rs-Lys2) using two sets of gene-specific primers [21]: Lys1-F2RT (CCCGTACCAAATAGCAAGAG) and Lys1-R2RT (TGTTAAGACCCGGCCCTGG) for Rs-Lys1 and Lys2-tF1 (TGCGTTTTACTCTCGTCTTCAA) and Lys2-tR1 (ACTGAACATTGACTACGTGAGAG) for Rs-Lys2. These were sequenced in both directions (Big Dye Terminator cycle sequencing, electrophoresis on ABI 3100, Applied Biosystems, Foster City, CA). Consensus sequences were aligned using the Clustal X algorithm [32] from the BioEdit v. 7.0.4.1 sequence editor [33] and corrected manually.

### Western Blot Analysis

The protein samples extracted from worker salivary glands (SG), worker legs, immature eggs dissected from queen ovaries (IME), queen accessory glands (AG) and eggs collected from termite nests were separated on 15% SDS-polyacrylamide gels. The amount applied for each protein was 80 µg (20-worker equivalent [eq.]), 100 µg (10-worker eq.), 2.0 mg (0.25-queen eq.), 10 µg (0.25-queen eq.) and 0.5 mg (80-egg eq.), respectively. In addition, 1.5 µg of hen egg lysozyme (HEL) was applied as a positive control. The peptide molecular weight marker DAIICHI (Daiichi Pure Chemical Co., Ltd., Tokyo, Japan) was used as a protein molecular weight standard. After electrophoresis, proteins were electrotransferred onto the Immun-Blot PVDF membranes (Bio-Rad Laboratories, Hercules, CA) with the Mini Trans-Blot Electrophoretic Transfer Cell (Bio-Rad Laboratories) following the manufacturer's instructions. After transfer, membranes were stained with Coomassie brilliant blue R-250 (CBB), and then washed and incubated with blocking buffer (2% ECL Advance Blocking Agent [Amersham Biosciences], 1× PBS [pH 7.6], 0.1% Tween 20) for 60 min at room temperature. The primary antibody was 50 µg of the rabbit anti-lysozyme polyclonal antibody NE098/7S (Nordic Immunological Laboratories, Tilburg, the Netherlands) per ml diluted in blocking buffer. Membranes were subsequently treated with the ECL horseradish peroxidase-linked donkey anti-rabbit IgG (GE Healthcare) at a 1∶1000 dilution for 60 min at room temperature. Protein binding was detected using the ECL Advance Western Blotting Detection Kit (Amersham Biosciences) and exposed to Polaroid film with the ECL mini-camera.

## Supporting Information

Figure S1 Gel filtration column chromatography elution patterns of the TERP. The active peak of the TERP is indicated by an arrow (left). Lysozymes readily bound with coexisting proteins because of the high isoelectric point. After severe precipitations, the active peak was not detected (right), and thus the sample lost egg recognition activity.
(1.37 MB EPS)

Figure S2 The bacteriolytic activity of termite lysozyme. a, Clearly visible inhibition area around a paper disc (5 mm in diameter; Advantec Toyo, Tokyo) containing 3 mg of termite egg extract in the LB agar plate colonised with the gram-positive bacteria Bacillus subtilis (NBRC 3009) after 48 h incubation at 37°C. b, Lytic activity of termite salivary lysozyme (TSL) and termite egg lysozyme (TEL). Data are means and standard deviations from two replicates. The mean percentage of the decrease in absorbance at 450 nm at 6 h (TSL) and at 24 h (TEL) after mixing was compared with the control using a two-tailed t-test. ***: P<0.001. Data were arcsine-root-transformed before statistical analysis.
(4.17 MB EPS)

Figure S3 Comparison of egg desiccation between eggs kept with and without egg grooming by workers. Twenty eggs were maintained in a 35-mm Petri dish either with or without 20 workers at 25°C and 60% humidity for 5 h. The weight loss of 20 eggs was measured to 0.01 mg using an electrobalance. Data are means and standard errors from five replicates. Workers frequently groomed eggs and supplied water, significantly reducing weight loss from desiccation (Man-Whitney U-test, Z = 2.61, P<0.01).
(0.57 MB EPS)

Figure S4 Phylogenetic relationship based on the amino acid sequences of c-type lysozymes obtained using the neighbour-joining method. Complete amino acid sequences of c-type lysozymes were obtained from the NCBI database. Accession numbers are indicated in parentheses. Bootstrap values are indicated for nodes having >50% support (500 replicates). Black arrows indicate termite lysozymes and hen egg lysozyme (HEL) is indicated by a red arrow.
(1.50 MB EPS)

Table S1 Cross-species activity of egg recognition
(0.06 MB DOC)

Movie S1 Egg grooming behavior of termite workers in a nursery cell. Workers pick eggs from the egg pile, groom them and then return them into the egg pile. Eggs were coated with saliva and thus protected against desiccation and pathogenic infection.
(4.38 MB MPG)
