# Supplementary material for: The Antibacterial Protein Lysozyme Identified as the Termite Egg Recognition Pheromone
Source: PLoS One. 2007 Aug 29;2(8):e813. doi: 10.1371/journal.pone.0000813 (PMC1950569; doi:10.1371/journal.pone.0000813)
Supplement: Table S1 — Cross-species activity of egg recognition (0.06 MB DOC) [file pone.0000813.s005.doc]

**Table S1:** **Cross-species activity of egg recognition**

|  | | Worker | | | | | | | | | | | | |
| --- | --- | --- | --- | --- | --- | --- | --- | --- | --- | --- | --- | --- | --- | --- |
|  |  | *Rs* | *Rk* | *Ra* | *Rm* | *Ry* | *Ro* | *Rh* | *Rv* | *Rf* | *Cf* | *Gf* | *Zn* | *Nt* |
| Egg | *Rs* | +++ | +++ | +++ | +++ | +++ | +++ | +++ | +++ | +++ | + | − | n | − |
|  | *Rk* | +++ | +++ | +++ | +++ | +++ | n | +++ | +++ | +++ | n | − | n | n |
|  | *Ra* | +++ | +++ | +++ | +++ | +++ | n | +++ | +++ | +++ | n | − | n | n |
|  | *Rm* | +++ | +++ | +++ | +++ | +++ | n | +++ | +++ | +++ | n | − | n | n |
|  | *Ry* | +++ | +++ | +++ | +++ | +++ | n | +++ | +++ | +++ | n | − | n | n |
|  | *Ro* | +++ | n | n | n | n | +++ | n | n | n | n | − | n | n |
|  | *Rh* | +++ | +++ | +++ | +++ | +++ | n | +++ | +++ | +++ | n | − | n | n |
|  | *Rv* | +++ | +++ | +++ | +++ | +++ | n | +++ | +++ | +++ | n | − | − | n |
|  | *Rf* | +++ | +++ | +++ | +++ | +++ | n | +++ | +++ | +++ | n | − | − | n |
|  | *Cf* | n | n | n | n | n | n | n | n | n | n | n | n | n |
|  | *Gf* | − | − | − | − | − | − | − | − | − | n | +++ | n | n |
|  | *Zn* | n | n | n | n | n | n | n | − | − | n | n | +++ | n |
|  | *Nt* | n | n | n | n | n | n | n | n | n | n | n | n | n |

Twenty eggs of each species were randomly arranged on moist unwoven cloth in a 35-mm Petri dish and kept with 10 workers for 24 h at 25°C. This bioassay was replicated three times for each combination. Mean piled-up rates were evaluated as −: < 25%, +: > 25%, ++: > 50%, +++: > 90%, n: not tested. *Rs*: *Reticulitermes speratus* (collected in Okayama city, Okayama, Japan), *Rk*: *R. kanmonensis* (Onoda, Yamaguchi, Japan), *Ra*: *R. amamianus* (Amami-Oshima Is., Kagoshima, Japan) *Rm*: *R. miyatakei* (Amami-Oshima Is., Kagoshima, Japan), *Ry*: *R. yaeyamanus* (Iriomote Is, Okinawa, Japan), *Rh*: *R. hageni* (Duke, North Carolina, USA), *Rv*: *R. virginicus* (Raleigh, North Carolina, USA), *Rf*: *R. flavipes* (Raleigh, North Carolina, USA), *Cf*: *Coptotermes formosanus* (Shirahama, Wakayama, Japan), *Gf*: Glyptotermes fuscus, *Zn*: *Zootermopsis nevadensis* (Santa Cruz, California, USA), *Nt*: *Nasutitermes takasagoensis* (Ishigaki Is. Okinawa, Japan).
